# DNA methylation-histone modification relationships across the desmin locus in human primary cells

**DOI:** 10.1186/1471-2199-10-51

**Published:** 2009-05-27

**Authors:** Marianne Lindahl Allen, Christoph M Koch, Gayle K Clelland, Ian Dunham, Michael Antoniou

**Affiliations:** 1 Nuclear Biology Group, King's College London School of Medicine, Department of Medical and Molecular Genetics, 8th Floor Tower Wing, Guy's Hospital, London SE1 9RT, UK; 2 The Wellcome Trust Sanger Institute, Wellcome Trust Genome Campus, Hinxton, Cambridge, UK; 3 Current address : Department of Biological Chemistry & Molecular Pharmacology, Harvard Medical School, 240 Longwood Avenue, Boston, MA, USA; 4 Current address : QIAGEN Hamburg GmbH, Koenigstr. 4 a, 22767 Hamburg, Germany; 5 Current address : EMBL-European Bioinformatics Institute (EBI), Wellcome Trust Genome Campus, Hinxton, Cambridge, UK

## Abstract

**Background:**

We present here an extensive epigenetic analysis of a 500 kb region, which encompasses the human desmin gene (*DES*) and its 5' locus control region (LCR), the only muscle-specific transcriptional regulatory element of this type described to date. These data complement and extend Encyclopaedia of DNA Elements (ENCODE) studies on region ENr133. We analysed histone modifications and underlying DNA methylation patterns in physiologically relevant *DES *expressing (myoblast/myotube) and non-expressing (peripheral blood mononuclear) primary human cells.

**Results:**

We found that in expressing myoblast/myotube but not peripheral blood mononuclear cell (PBMC) cultures, histone H4 acetylation displays a broadly distributed enrichment across a gene rich 200 kb region whereas H3 acetylation localizes at the transcriptional start site (TSS) of genes. We show that the *DES *LCR and TSS of *DES *are enriched with hyperacetylated domains of acetylated histone H3, with H3 lysine 4 di- and tri-methylation (H3K4me2 and me3) exhibiting a different distribution pattern across this locus. The CpG island that extends into the first intron of *DES *is methylation-free regardless of the gene's expression status and in non-expressing PBMCs is marked with histone H3 lysine 27 tri-methylation (H3K27me3).

**Conclusion:**

Overall, our results constitute the first study correlating patterns of histone modifications and underlying DNA methylation of a muscle-specific LCR and its associated downstream gene region whilst additionally placing this within a much broader genomic context. Our results clearly show that there are distinct patterns of histone H3 and H4 acetylation and H3 methylation at the *DES *LCR, promoter and intragenic region. In addition, the presence of H3K27me3 at the *DES *methylation-free CpG only in non-expressing PBMCs may serve to silence this gene in non-muscle tissues. Generally, our work demonstrates the importance of using multiple, physiologically relevant tissue types that represent different expressing/non-expressing states when investigating epigenetic marks and that underlying DNA methylation status should be correlated with histone modification patterns when studying chromatin structure.

## Background

Epigenetic modifications that affect gene expression include post-translational modifications on N-terminal tails of histones and DNA methylation. Detailed analysis of epigenetic status can provide insight into the location and function of genetic regulatory elements on a genome-wide basis. A major challenge in the post-genomic era is the mapping and functional characterization of the full complement of transcriptional regulatory elements including promoters, enhancers, locus control regions (LCRs) and chromatin domain boundary elements. As a first step in meeting this objective, the **Enc**yclopaedia **O**f **D**NA **E**lements (ENCODE) project was launched, which aims to map the complete set of genetic control elements within selected regions amounting to 1% (~30 Mb) of the human genome [1-4].

The human desmin gene (*DES*) locus (ch2q35) is covered in part within one of the ENCODE regions designated as ENr331, which was chosen due to its high gene density and high non-exonic sequence conservation. ENr331 covers approximately 500 kb of chromosome 2q35 (hg 17 co-ordinates chr2:220,102,851–220,602,850; assembly May 2004) and includes *DES *and the downstream genes but excludes its locus control region (LCR) [5,6]. The *DES *LCR is the only muscle-specific transcriptional regulatory element of this type described to date and constitutes a highly evolutionarily conserved element located between 9–18 kb 5' of *DES *that has been shown to drive reproducible, full physiological levels of expression in all muscle cell types [5,6]. As in the case of β-globin gene clusters [7], the *DES *LCR may co-ordinate expression of some or all of the downstream muscle-specific genes within this region namely *DES, APEG1, SPEG *and *CHPF*. This type of gene/regulatory element configuration has been referred to as a functional gene domain [8,9]. The 5' end of *DES *is also associated with a small compact methylation-free CpG island [10] that spans the first exon, a common feature for approximately 40% of tissue specifically expressed genes [11].

Several post-translational histone modifications have now been identified which affect regulation of gene expression such as lysine acetylation/methylation and phosphorylation on serine residues [12]. With advances in detection methods for these modifications, subtle differences between varying histone marks have been described. For example, histone H3 and H4 acetylation demonstrate a slightly different pattern throughout the *GH1 *[growth hormone GH-N, 13] and the *HBB_CHICK *[14,15] gene clusters both of which contain LCR elements. Enhancers and other types of transcriptional regulatory elements can be predicted through a determination of their histone modification pattern, which appears distinct from that observed at promoter or TSS regions. It is now clear that certain histone patterns are highly predictive of the transcriptional state of DNA [3,4].

Eukaryotic DNA can be methylated at the cytosine of a CpG dinucleotide, which generally has a repressive effect on transcription [16]. There is strong evidence to suggest a link between DNA methylation status and certain repressive histone modification marks [17-19] particularly methylation of histone H3 at lysine 9 (H3K9) and 27 (H3K27), which are important modifications for gene repression [20]. Furthermore, tri-methylation of histone tail H3 lysine (K) 27 (H3K27me3) [21], has been shown to preferentially mark unmethylated DNA in early development and cause silencing by recruiting repressive Polycomb group (PcG) complexes [18].

We have therefore undertaken an analysis of both DNA methylation and histone modifications to gain greater insight into the types of epigenetic marks that are functionally correlated with the *DES *locus and especially at the *DES *LCR. As the epigenome will vary between different tissues displaying different gene expression profiles, it is important to conduct such epigenetic investigations in an appropriate range of cell types that will represent expressing and non-expressing states for a genomic region of interest. Recent work has addressed this issue to some extent [4] but here we strive to give a more detailed and focused analysis of a well characterized gene locus in primary human cells which are more physiologically relevant.

We have employed a wide range of techniques to substantially extend the epigenetic analysis of ENr331 in primary muscle and non-muscle human cell types to provide insight into which types of histone modifications can co-exist at a given locus with a corresponding underlying DNA methylation pattern. Our results show that in expressing skeletal myoblast/myotube cultures generally histone H4 acetylation is broadly distributed across a gene rich 200 kb region of chromosome 2q35 encompassed within ENr331 extended to include the *DES *LCR whereas H3 acetylation localizes at the TSS of genes. More specifically, the *DES *LCR and first exon are enriched with hyperacetylated domains of histone H3 and that H3K4 me2 and me3 exhibit a different distribution pattern across this locus. In addition, CpG methylation combined with histone mapping identified one of the DNaseI hypersensitive site (HS) elements that constitute the *DES *LCR as of potential critical functional importance. We found the CpG island spanning the *DES *promoter to be unmethylated regardless of expression status and to be marked with H3K27me3 in PBMCs, which may serve a promoter silencing role in non-muscle cells.

## Results

### ChIP-on-chip: global H3 and H4 histone acetylation patterns across 500 kb of chromosome 2q35 encompassing the *DES *locus

The *DES *locus contains three muscle specific/preferentially expressed genes, *DES APEG1 *and *SPEG*, which are flanked by two housekeeping genes *DNPEP *and *GMPPA*. This region also includes the *DES *LCR which lies 9–18 kb 5' of *DES *and which is composed of 5 muscle-specific DNaseI HS numbered 1–5 in a 3' to 5' direction [6]. Analysis of the DNA sequence that underlies the elements of the *DES *LCR shows sub-regions of high (83–100%) sequence conservation between rodents (mouse/rat) and humans within or near each HS site designated with the letters a, b, c or d [6]. Expression of *DES *increases upon fusion of unfused (UF) skeletal myoblasts to fused (F) myotubes [22] (see Additional file 1, Figures S1 and S2).

**Figure 1:**
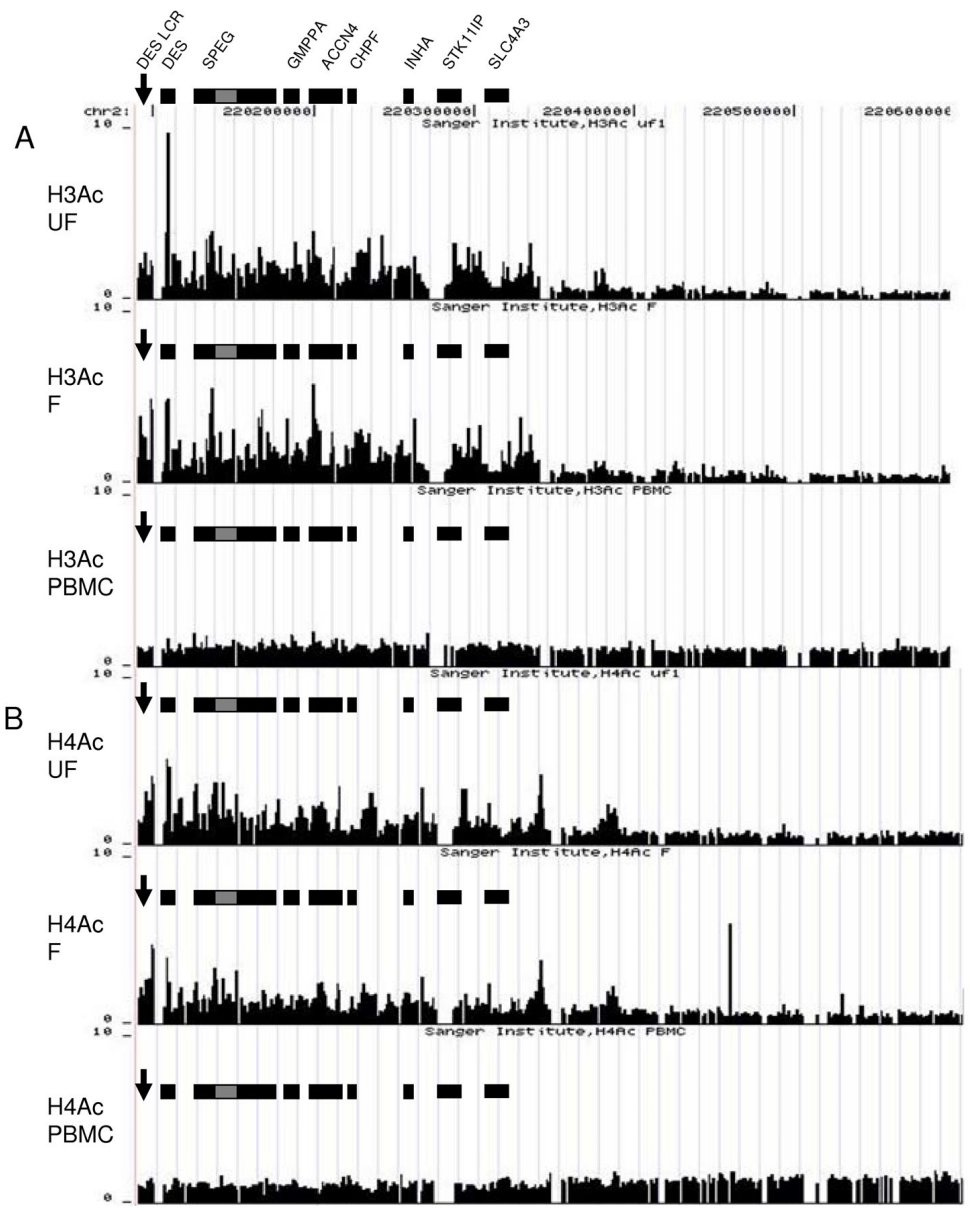
**Microarray analysis for histone H3 and H4 acetylation across the *DES *LCR and ENCODE region ENr331 covering 500 kb of human chromosome 2q35**. Chromatin (mono-tetranucleosomes) prepared using the nChIP approach was hybridized to a microarray tile, which covers a 500 kb region of chromosome 2q35 and encompasses the *DES *LCR and *DES*. Annotated genes taken from the UCSC genome browser  are shown as black rectangles in each panel. **(A) **A peak of histone H3 acetylation can be observed at the beginning of the genes *DES*, *SPEG *and *ACCN4*. Note the grey bar indicating the position of *APEG1*. **(B) **Histone H4 acetylation appears over the entire gene rich region and is highly enriched across the *DES *promoter and LCR in muscle cells. The solitary peak of H4 acetylation downstream of the gene rich region (Panel F) was caused by an excess of Cy3 dye background staining on the corresponding part of the slide and therefore does not represent a true enrichment. Fold enrichment was obtained from an average ratio of two independent experiments.

The ENCODE region ENr331 which spans 500 kb of chromosome 2q35 does not include the *DES *LCR as it terminates -5.7 kb 5' of *DES*. However, ENr331 does span *DES *and the more downstream genes *SPEG, GMPPA, ACCN4, CHPF, INHA, SLC4A3 *(Figure 1). A small spotted microarray consisting of ~500 bp overlapping PCR products was designed to extend the area covered by ENr331 to encompass the *DES *LCR (-18 kb to -8.3 kb 5' of the *DES *TSS). Note: the PCR primers used to extend the region of the array are shown in Additional file 1, Table ST1.

Native chromatin without cross-linking, with a size range of mono-tetranucleosomes was isolated from cultures of human primary myoblasts, myotubes and PBMCs and subjected to immunoprecipitation with antibodies against histones H3 and H4 acetylated at multiple lysine residues, namely lysines 9 and 14 on H3 and lysines 5, 8, 12 and 16 on H4, which have been associated with a transcriptionally competent chromatin state [23,24]. This allowed a complete analysis of the histone H3 and H4 acetylation pattern of this entire area in unfused (UF) myoblasts, fused (F) myotubes and PBMCs (Figure 1).

The peaks of enrichment of H3 lysine acetylation throughout the region analysed corresponded to the TSS of most of the genes including *DES *(Figure 1A). The *DES *LCR was enriched for H3 acetylation approximately 2–5 fold above non-muscle PBMCs in both myoblasts and myotubes (Figure 1A and Additional file 1, Figure S3).

In contrast to H3 acetylation, which exhibits a relatively tight peak of enrichment localized to the *DES *promoter, H4 acetylation displays abundance across the entire transcribed region of *DES *(Figure 1B; panels UF and F) and extends into intergenic areas. This was also the case with other individual genes within the region such as *CHPF*, which exhibited a broad enrichment across its entire gene body (Figure 1B and Additional file 1, Figure S3). A ~5-fold enrichment of H4 acetylation was observed at the *DES *LCR and promoter compared with PBMCs (Figure 1B and Additional file 1, Figure S3). Similarly to H3 acetylation, there was very little enrichment of histone H4 acetylation in PBMCs across the area analysed (Figure 1; panels PBMC). See Additional data file 1, Figure S3 and S3' for detailed analysis of individual genes with the region.

The current genome browser build hg18 only illustrates *SPEG*. However it must be noted that *APEG1 *is one of its isomers and is transcribed from an internal separate promoter [6]. The presence of the internal (*APEG1*) promoter accounts for the presence of a peak of acetylation seen on the microarray at this position (Figure 1 and Additional file 1, Figure S3).

### *DES *and the *DES *LCR show high levels of tissue-specific but a selective histone modification pattern

Having obtained a general overview of histone H3 and H4 acetylation patterns across the *DES *locus and surrounding 500 kb region, we next sought to obtain a more detailed map of epigenetic status specifically across the *DES *LCR, as this constitutes a crucial regulatory element. Chromatin used for the microarray analysis was analysed by Q-PCR to assess enrichment for selected regions within *DES *and the *DES *LCR.

**Figure 2:**
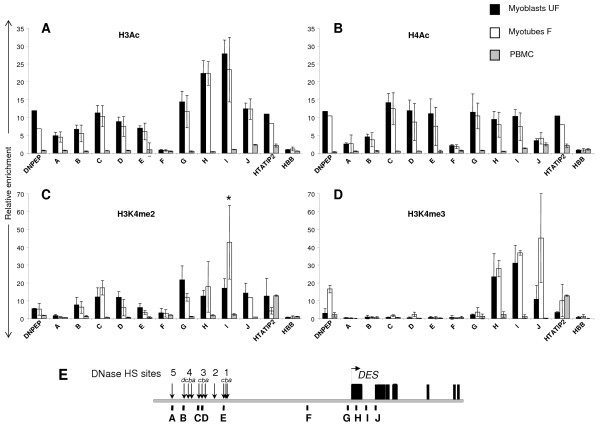
**Fine resolution mapping of histone modifications across the *DES *LCR and *DES *in myoblasts and myotubes compared to PBMCs**. Chromatin was prepared from myoblasts, myotubes and PBMCs for nChIP (as Figure 1) and amplified using Q-PCR to gain a comprehensive picture of histone modifications status across the *DES *LCR and *DES*. **(A) **Histone H3 acetylation in unfused myoblasts (UF, black bars), in fused myotubes, which express *DES *2-fold higher than myoblasts (F, white bars) and in non-expressing PBMCs (grey bars) across *DES *and the *DES *LCR. **(B) **As panel (A) but showing the distribution of histone H4 acetylation. **(C) **Histone H3K4 di-methylation (H3K4me2) in unfused myoblasts (UF, black bars), in fused myotubes (F) and in non-expressing PBMCs (grey bars) across *DES *and the *DES *LCR. **(D) **As panel (C) but showing histone H3K4me3 enrichment across *DES *and the *DES *LCR. Positive controls include the housekeeping genes *DNPEP *and *HTATIP2 *and as a non-expressing negative control, *HBB*. **(E) **Illustration of *DES *and *DES *LCR genomic region. The black rectangles and horizontal arrow denote the exons and direction of transcription of *DES *respectively. Vertical arrows indicate the relative positions of the *DES *LCR HS and evolutionarily conserved regions. Vertical bars designated A-J denote the positions of the PCR amplicons following nChIP. Results are presented as fold-enrichment of IP samples over input DNA. Error bars represent the standard deviation about the mean fold-enrichment from at least 2 separate PCRs performed in duplicate from 2 independent nChIP experiments. Enrichment across the muscle-specific *DES *locus was above non-specific binding background controls (no antibody and IgG) in the myoblast/myotube cultures but not PBMCs. P value < 0.05 denoted by an asterisk (*).

Histone H3 and H4 acetylation showed a marked enrichment of between 5 to 15-fold above PBMC levels in both myoblasts and myotubes at all of the positions tested within the *DES *LCR (Figure 2A and 2B; amplicons A-E). Element HS3c exhibited the highest overall level of histone acetylation within the LCR in both myoblasts and myotubes reaching levels of ~15-fold enrichment (Figure 2A and 2B; amplicon C). We also observed enrichment of H3 acetylation across the *DES *TSS (Figure 2A; amplicons G-J) but with the highest level present 3' of the first exon at position +0.8 kb (Figure 2A; amplicon I). This level of enrichment decreased by almost 2-fold at the second exon (Figure 2A; amplicon J). Histone H4 acetylation was enriched at similar levels (~10–15 fold) across parts of the LCR and *DES *(Figure 2B; amplicons C-E and G-J).

PBMCs did not show any enrichment of histone H3 and H4 acetylation across either *DES *or its LCR reflecting these elements muscle-specific nature and function (Figure 2A and 2B, amplicons A-J). The only enrichment of both histone H3 and H4 acetylation in the PBMC sample was seen at the TSS of *DNPEP *and *HTATIP2*, which is as expected for ubiquitously expressed genes (Figure 2A and 2B; amplicons *DNPEP *and *HTATIP2*). We also investigated the chromatin structure between HS1 of the LCR and the *DES *TSS, a region at a position approximately -4 kb 5' of *DES *and found no enrichment of histone H3 or H4 acetylation in any of the samples (Figure 2A and 2B; amplicon F).

The microarray data correlates well with the higher resolution native ChIP (nChIP) results both showing similar trends in H3 and H4 acetylation. However, the Q-PCR and microarray sets of data are technically very different and therefore quantitative comparisons between the two are not possible.

### Histone H3K4me2 marks the *DES *LCR

Histone methylation patterns have been shown to mark different regions of a transcribing gene and to distinguish between promoters and distal enhancers [3,25]. We therefore assessed the presence of histone H3K4me2 and H3K4me3 at the same regions analysed for global H3 and H4 histone acetylation (Figure 2A and 2B). This also allowed the correlation between histone acetylation and methylation to be examined. H3K4me2 displayed a widespread distribution across the region being enriched up to 20-fold above background levels at elements HS4d to HS1 within the *DES *LCR (Figure 2C; amplicons B-E). The unfused myoblasts exhibited a 20-fold enrichment across the first 2 kb of *DES *(Figure 2C; amplicons G-J) whereas myotube cultures showed the same level of enrichment but had a high peak of ~40-fold of H3K4me2 at the end of exon 1 of *DES *(Figure 2C; amplicon I). PBMCs did not show any enrichment across the *DES *LCR or *DES *but showed a marked abundance of ~25-fold above background of H3K4me2 at the control housekeeping gene promoter of *HTATIP2 *as expected from previous work [26].

H3K4me3 was not enriched at any site within the *DES *LCR within any of the cell types tested (Figure 2D; amplicons A-E). However, there was a ~40-fold enrichment of this mark above background levels before the start of the second exon of *DES *in both myoblasts and myotubes (Figure 2D; amplicons H-J). This decreased sharply in the myoblast sample by the second exon compared to that observed in the myotube culture, which remained high at this position (Figure 2D; amplicon J).

### DNA methylation status of CpG sites within *DES *and *DES *LCR

**Figure 3:**
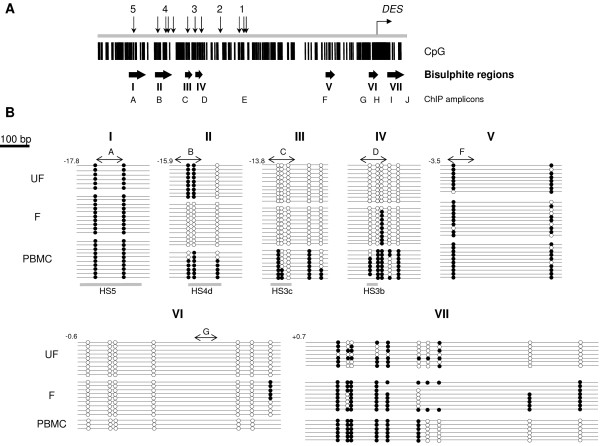
**High-resolution DNA methylation mapping of individual CpG dinucleotides within highly conserved regions of the *DES *LCR**. Sodium bisulphite conversion of *Hind*III-digested human unfused myoblast (UF), fused myotube (F) and PBMC DNA followed by PCR amplification, cloning and sequencing was used to obtain a high-resolution map of the methylation status across GC-rich regions of high sequence homology between mouse, rat and human within the *DES *LCR. **(A) **Top line: illustration of the *DES *and *DES *LCR HS 1–5 and positions of the evolutionarily conserved elements (vertical arrows). Middle line: CpG density map. Each vertical line represents a single CpG dinucleotide residue. Bottom line: position of PCR amplicons following genomic DNA bisulphate conversion and nChIP (Figure 2). **(B) **Methylation status of individual CpG sites. Each line represents the result within independent clones of PCR amplified regions. Open/white circles indicate an unmethylated CpG site; closed/black circles, methylated CpG sites. At certain sites circles are missing, sequence data was not available for these individual CpG sites. Amplicons used for nChIP analysis are shown (A-G) by a horizontal double-ended arrow. Positions of sequenced regions relative to the TSS of *DES *are indicated in kb. Regions of homology within the LCR are marked by a horizontal grey line.

*DES *is approximately 8.3 kb in length with 9 exons and is associated with a small compact CpG island of 550 bp that spans its first exon and has a CpG count of 74, a CpG percentage of 27.0% and a ratio of observed to expected CpG dinucleotides of 1.08. Due to the tissue-specific expressed nature of this gene we were able to correlate the histone modification patterns we observed within *DES *and the *DES *LCR with the underlying DNA methylation pattern in different transcriptionally active and inactive states. DNA methylation was analysed using sodium bisulphite conversion coupled with PCR/sequencing in adult human primary myoblasts, myotubes and PBMCs at CpG rich areas throughout the LCR and *DES *(Figure 3; amplicons I-VII) and which corresponded to regions of high sequence conservation between rodents (mouse/rat) and human [6].

Regions analysed within the *DES *LCR included HS5 and HS4d (Figure 3; amplicons I and II). The two CpG sites within HS5 within the region of rodent/human homology were fully methylated in all the cell types analysed (Figure 3B; panel I). HS4d contained 3 CpG dinucleotides all of which were unmethylated in the myotubes (Figure 3B; panel II, F), showed a mixed pattern of methylation in myoblasts (Figure 3B; panel II, UF) but were ~96% methylated in PBMCs (Figure 3B; panel II, PBMCs).

Within element HS3 only sub-regions HS3c and HS3b were analysed, as HS3a does not contain any CpG dinucleotides. All the CpG sites within HS3c and b in the unfused myoblasts were unmethylated (Figure 3, panel III and IV; UF). Within HS3b in the myotubes the majority of the CpG sites were unmethylated apart from 1 out of the total of 5 CpG sites analysed, which exhibited 90% methylation (Figure 3B, panel IV; F). All the CpG sites except for one within HS3c showed a high percentage of methylation (~40–100%) in PBMCs (Figure 3B, panel III and IV; PBMC) demonstrating that this region is highly methylated compared to muscle cells.

The *DES *TSS genomic region was analysed to determine where the region of transition occurs between unmethylated and methylated DNA in both a 5' and 3' direction (Figure 3). The 10 CpG sites within the +0.7 kb-+1.5 kb region exhibit a mixed pattern of methylation in both muscle and non-muscle cell types (Figure 3B; panel VII), which indicates that the methylation-free CpG area of DNA appears to end +0.7 kb 3' of the *DES *TSS. Analysis of a region ~0.5 kb upstream of the *DES *TSS at the start of the CpG island, showed that all seven CpG sites in this area lack methylation (Figure 3B; panel VI) in all cell types including PBMCs. Therefore, this unmethylated DNA is not related to the transcriptional status of *DES*. The extent of the island is restricted to the GC dense region spanning the first exon of *DES *(Figure 3A; CpG). These results also demonstrate that the gene body of *DES *is methylated starting before the start of the second exon even in expressing muscle cell types (Figure 3B; panel VII). Gene body methylation has now been reported in several systems [27-30].

### The unmethylated CpG island spanning the *DES *promoter is marked by H3K27me3 in PBMCs

DNA methylation analysis showed that the CpG island spanning the *DES *promoter was unmethylated in both muscle and non-muscle cell types (Figure 3). Given that methylation-free CpG islands are usually associated with an open chromatin structure this poses the question as to how the *DES *promoter region is repressed in non-expressing cells? The absence of muscle specific transcription factors in non-muscle cells could be sufficient to prevent transcription of *DES *in non-muscle cells. However, the *DES *promoter does possess ubiquitous transcription factor binding sites such as Sp1 [31] that would be expected to be functional and therefore suggesting that additional mechanisms may contribute to the complete silencing of *DES *in non-muscle cells.

**Figure 4:**
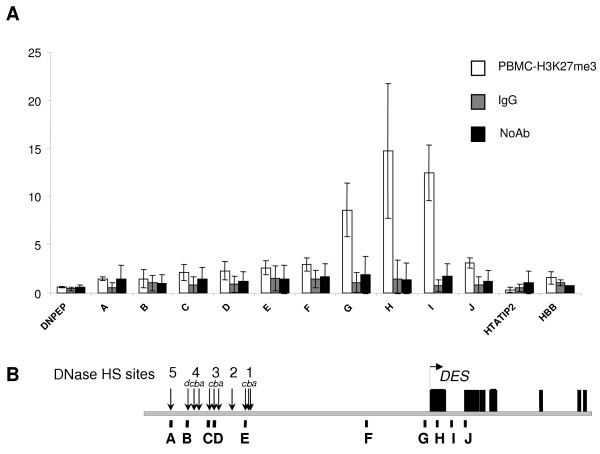
**Histone H3 lysine (K) 27 tri-methylation (me3) is highly enriched at the *DES *CpG island in non-expressing PBMCs**. nChIP experiments (Figure 2) were performed using antibodies against H3K27me3. **(A) **H3K27me3 of *DES *and the *DES *LCR in non-expressing PBMCs (grey bars). Non-specific binding was assessed by employing IgG (dark grey bars) and no antibody (stripped bars) samples as controls. Results are presented as fold-enrichment of IP samples over input DNA. Error bars represent the standard deviation about the mean fold-enrichment from at least 2 separate PCRs performed in duplicate from 2 independent nChIP experiments. **(B) **Illustration of *DES *and the *DES *LCR. Vertical arrows indicate the regions of high sequence conservation within each HS site of the LCR and the *DES *transcriptional start site (TSS) is represented by a horizontal arrow. Black rectangles denote the exons of *DES*. PCR amplicons (A-J) are shown by vertical black bars. Significant H3K27me3 enrichment was seen only at the *DES *promoter/CpG island.

A possible candidate epigenetic mark for suppressing expression from the *DES *promoter in non-muscle tissues is H3K27me3, which has been implicated in marking unmethylated DNA for silencing [18]. In addition, methylation of H3K27 has been universally linked to gene repression [21,32-34] unlike H3K9 methylation which has been found at both inactive and active genes [12,35,36]. Therefore, we determined whether H3K27me3 was present across the LCR and *DES *in non-muscle PBMCs and muscle cells. The only region that was found to be enriched by 9–15 fold for H3K27me3 above background levels was across the *DES *unmethylated CpG island in the PBMC sample (Figure 4; amplicons G-I). This peak of enrichment was tightly localized to the methylation-free CpG island and dropped to background levels by the second exon of *DES *(Figure 4; amplicon J). No significant enrichment above background was seen in the myoblast/myotube cultures (data not shown). Interestingly, this mark was not seen at either the -4 kb region between the LCR and the *DES *TSS or across the LCR in PBMCs (Figure 4; A-E). In addition, H3K27me3 was not observed at the CpG poor promoter of *HBB *that is also silent in PBMCs (Figure 4A). These observations provide strong support for the concept that H3K27me3 is a histone modification that marks silent chromatin at specific genomic locations [21].

## Discussion

Our work constitutes the first detailed epigenetic investigation of a muscle-specific LCR associated with *DES*. We have conducted an extensive epigenetic analysis of 500 kb of human chromosome 2q35, which includes the muscle-specific *DES *locus and encompasses ENCODE region ENr331. Our work expands on previous analysis of ENr331 on several points. Firstly, the microarray tile was extended to include the *DES *LCR, a crucial transcriptional regulatory element in this genomic region. Secondly, we have analysed the epigenetic status of ENr331 and especially the *DES *and *DES *LCR regions in human primary skeletal muscle cells, both proliferating myoblasts and post-mitotic differentiated myotubes as well as non-muscle PBMCs. Therefore, unlike previous investigations of this region we were able to compare epigenetic patterns in physiologically relevant, expressing/non-expressing situations. Thirdly, we have correlated histone modification patterns across the *DES *and *DES *LCR with the underlying DNA methylation pattern, again in expressing and non-expressing states of the locus.

Our results from microarray ChIP-on-chip studies show that in myoblast/myotube cultures the *DES *locus lies within an extended hyperacetylated chromatin region that was not present in non-muscle PBMCs (Figure 1) suggesting a role of the muscle specific *DES *LCR in establishing this active domain. The ChIP-on-chip results also highlight differences in histone acetylation between gene rich and gene poor areas over this 500 kb region.

High resolution nChIP coupled with Q-PCR was employed to validate the microarray data and dissect the histone modification patterns across the 5'region of *DES *including its LCR. The analysis showed that histone H4 acetylation displayed similar levels of enrichment across the LCR and the first 2 exons of *DES *in skeletal muscle cells (Figure 2B). This is in contrast to H3 acetylation, which was more abundant around the *DES *TSS than at the LCR (Figure 2A). The peak of H3 acetylation over the *DES *promoter and TSS may be accounted for by the presence of MEF-2 and E-box elements within these regions [31]. MEF-2 and the myogenic factors are known to recruit histone acetyltransferase (HAT) activity [37] and therefore their binding and action at the *DES *promoter and enhancer elements could produce the peak of H3 acetylation that we observe at this region.

Interestingly, unfused myoblasts and fused myotubes displayed similar histone modification and DNA methylation patterns across the *DES *locus. This implies that an open chromatin region already exists in unfused myoblasts although they exhibit a lower level of *DES *expression. Therefore, the histone acetylation seen in myoblasts may have been established at an earlier time point before myoblast differentiation. Consequently, the increase of *DES *expression observed in myotubes could result from an enhancement in transcription factor binding activity rather than a change in overall chromatin architecture. The only significant difference between myoblast and myotube modification patterns that we observed was in histone methylation at *DES *3' of the first exon where in fused myotubes the levels of H3K4me2 and me3 are 20–30 fold higher than in the myoblasts (Figure 2C and 2D). This implies an increase in the levels of histone methyltransferase (HMT) activity across *DES *coupled with an increase of transcription.

DNA methylation analysis of the LCR showed that HS3 was unmethylated in skeletal muscle cells compared to PBMCs whereas HS4 and HS5 both contained methylated CpG sites even within the muscle cell samples (Figure 3). Interestingly, the mapped HS5 site lies within an Alu repeat element  which is also GC rich and may explain why this HS site is methylated. It has also been shown that the density of CpG dinucleotides is directly linked to their methylation state and that a low density of methylated CpG sites does not perturb transcription [28]. Therefore, in the case of HS5 which has a relatively small number of CpG sites their methylation status may not be relevant to its function.

Histone H3K27me3 has been correlated with inactive genes [21,32]. Here we show that, H3K27me3 marks the CpG island of *DES *only in non-expressing PBMCs (Figure 4) and this could constitute a mechanism by which the DNA at the CpG island promoter of *DES *remains unmethylated but inactive in all cells types except muscle. There are extensive genome wide studies on the histone patterns across genes and regulatory elements [3,21,32,34,38,39]. Our results are in concordance with previous genome wide analysis in particular H3K4me3 preferentially marking the *DES *TSS and H3K4me2 being more dominant at its regulatory element, the LCR. In addition, our data appear to be typical for complex regulatory elements of this type [14,15,36]. However, genome wide studies in the cell types used would not have illustrated the histone modification pattern across the muscle-specific *DES *locus, therefore our analysis shows the importance of using the appropriate cell type when analysing a tissue specific locus.

## Conclusion

Taken together these findings show that the *DES *gene cluster lies within a hyperacetylated transcriptionally competent muscle specific gene domain and that H3K4me2 and H3K4me3 mark the LCR in different distributions. In addition, H3K27me3 may contribute to the silencing of tissue-specific methylation-free CpG islands in non-expressing tissues. These observations provide insight into the important elements such as HS3 within the *DES *LCR and highlight the significance of also investigating the underlying DNA methylation pattern when examining chromatin structure at any given gene locus.

## Methods

### Mammalian Cell culture

Adult human primary myoblast cells were a generous gift from Serge Braun, Association Française contre les Myopathies, Evry, France (formerly at Transgene SA, Strasbourg, France) and were isolated from a skeletal muscle biopsy obtained after informed consent and in accordance with local French regulatory approval from a healthy 14 year old Caucasian male. These myoblast cells were cultured in Dulbecco's modified eagles medium (DMEM) supplemented with 10% fetal calf serum (FCS), 2% Ultroser^® ^G (Pall BioSepra, Cergy, France), 5 ml penicillin/streptomycin (pen/strep100 μg/ml) and 5 ml glutamine. To obtain fused myotubes, myoblasts were grown to confluency and transferred to fusion media consisting of DMEM, 10% FCS, 5 ml pen/strep (100 μg/ml), and 5 ml glutamine.

Whole blood samples were obtained by informed consent from normal healthy human volunteers in accordance with King's College London Ethical Committee guidelines and procedures. Whole blood was cultured for 72 hrs in 500 ml of RPMI (Roswell Park Memorial Institute) 1640, 5 ml 1 mg/ml *Phaseolus Vulgaris Phytohemagglutinin *(PHA-P 1 mg/ml, St. Louis, MO, USA), 5 ml pen/strep (100 μg/ml) and 5 ml glutamine. Peripheral blood mononuclear cells (PBMCs) were then isolated after lysis of red blood cells with RBC lysis buffer (0.1 mM EDTA, 155 mM NH_4_Cl, 10 mM KHCO_3_).

### Native Chromatin Immunoprecipitation (nChIP)

nChIP experiments were performed at least twice using polynucleosomes from independently prepared chromatin preparations as previously described [30]. Both ChIP-on-chip and Q-PCR was conducted on the same input material. ChIP analysis for various histone modifications were conducted as before [30]. Equivalent amounts of recovered nChIP input and immunoprecipitated (IP) DNA were analysed by quantitative real-time PCR (Q-PCR) using the QuantiTect SYBR Green PCR Master Mix (Qiagen, Crawley, UK) system on a LightCycler™ 1.2 (Roche Applied Science, Lewes, UK) as described [40]. Primers used for PCR amplification are shown in Additional file 1, Table ST1. A melting curve program was performed and showed that all PCR primers were specific for the target sequences (Additional data file 1, Figures S4B and B'). Fold enrichment of selected regions of DNA within IP samples was calculated by subtracting the IP Ct (cycle threshold) value from the input Ct value and was presented as a ratio of 2^-CtIP ^to 2^-Ct input ^[30,40]. All primers were optimized to amplify the same amount of DNA with equivalent efficiency (Additional data file 1, Figures S4C and C').

#### Antibodies

The following antibodies were used: 20 μg of anti-Histone H3 acetylation lysines (K) 9 and 14 (Millipore #06–599; Billerica, MA, USA), 50 μl of anti-Histone H4 acetylation lysines (K) 4,7,11 and 14 (Millipore, #06–866), 50 μl of anti-Histone H3 dimethyl Lysine 4 (H3K4me2; Millipore, #07–030), 50 μl of anti-Histone H3 trimethyl Lysine 4 (H3K4me3; Millipore, #07–473) and 20 μl of anti-Histone H3 trimethyl Lysine 27 (H3K27me3: Millipore, #07–449).

### ChIP-on-chip analysis

A high resolution DNA microarray was constructed across 500 kb of chromosome 2 using 500 bp overlapping PCR products as previously described [41]. Briefly, primer pairs were designed using the Primer3 software and Web site with Repeat Masking. All primers had a universal amino-linked tag sequence (5'-TGACCATG-3') attached to the 5' end of the sense strand primer, to allow covalent binding to the microarray slide. PCR reactions were conducted on a Flexigene Techne PCR machine using BAC clone accession number AC053503 DNA as a template which encompasses the *DES *locus and surrounding regions. DNA was denatured at 94°C for five minutes followed by 30 cycles of 94°C for 30 seconds, 65°C for 30 seconds and 72°C for 60 seconds. A final extension step at 72°C for five minutes was performed. A 5 μl aliquot of the reaction was run on a 2.5% agarose gel to confirm product specificity. Amino-liked specific PCR products were spotted onto the glass array slides in duplicate by the microarray facility at the Wellcome Trust Sanger Institute, Hinxton, Cambridge.

Approximately 300 ng of nChIP DNA sample, input and IP were labelled using Cy-5 and Cy-3 dye modified dCTP respectively. These samples were pooled precipitated with human Cot-1 DNA (Roche Applied Sciences, Lewes, UK) and resuspended in 100 μl of hybridization buffer (10 ml 20×SSC, 50 ml deionised formamide, 10 ml 100 mM Tris-HCl pH 7.4, 5 g dextran sulphate, 100 μl TWEEN 20) and left at 65°C to allow the labelled DNA pellets to dissolve.

The samples were loaded onto the microarray slides in the Tecan HS 400™ Pro hybridization machine (Tecan Group Ltd., San Jose, USA) for a 72 hour hybridization reaction which included subsequent washing steps.

Hybridised microarray slides were scanned on an Axon 4000B scanner (Molecular Devices, Concord, Canada). The photon multiplier tube (PMT) levels used for detection were adjusted and tailored for each individual array. Arrays were analysed using Genepix 4.0. Software (Molecular Devices, Concord, Canada). Single image TIFF files from Genepix 4.0 were imported into the 'Spot' program for analysis [42]. The spot file was then uploaded into a custom made Excel based macro, which gave average enrichment ratios for each region represented on the array. The DNA chip data was normalized over the entire microarray which not only contained the ENr331 region but also other ENCODE areas under investigation, specifically loci from chromosomes 10, 11 14, 16 and X. The array also had smaller tiles from chromosomes 2, 5, 7 and 9. This allows accurate comparison of the histone modification patterns between different cell types. Even though diverse patterns of histone modifications can be compared between different ChIP samples, absolute levels of histone H3 and H4 acetylation cannot be evaluated as antibody affinities will vary [43]. The ChIP-on-chip experiment was conducted in duplicate for each spot on the array and repeated with an independent biological sample. The average ratio for each set of duplicates was then calculated and the mean was taken between independent experiments.

### DNA bisulphite conversion and sequencing

Total genomic DNA (2 μg) from myoblasts, myotubes and PBMCs, was digested with 5 μl *Hind*III (100 units) and 5 μl 10× buffer in a reaction volume of 50 μl for 3 hrs at 37°C. The digested DNA was then denatured by adding 3 M NaOH to a final concentration of 0.3 M and incubated at 37°C for 15–20 min. The denatured DNA was added to 1.1 ml of pre-warmed freshly prepared sodium bisulphite solution (4.5 M sodium bisulphite, 2.5 mM hydroquinone, 0.225 M NaOH, pH5), covered with 200 μl of mineral oil and incubated for 5 hrs at 55°C in the dark. The bisulphite treated DNA was purified using the QiaexII gel extraction kit (Qiagen). The sample was then desulphonated by adding 3 M NaOH to a final concentration of 0.3 M and incubated at 37°C for 15–20 min. The sample was then neutralized by adding 6 M ammonium acetate to a final concentration of 3 M and DNA precipitated by adding 3 volumes of 100% ice-cold ethanol. PCR amplification of selected regions with nested pairs of primers (see Additional file 1, Table ST1) was carried out on the bisulphite converted DNA using the same conditions as above but for 35 cycles. PCR products were cloned into the pCR 2.1 TOPO vector (Invitrogen, Paisley, UK) and sequenced using M13 forward and reverse primers.

## Abbreviations

*DES*: human desmin gene; LCR: locus control region; ENCODE: Encyclopaedia Of DNA Elements; HS: DNaseI hypersensitive site; H3K4me2: H3 lysine 4 di-methylation; TSS: transcriptional start site; H3K27me3: H3 lysine 27 tri-methylation; PBMCs: peripheral blood mononuclear cells; nChIP: native chromatin immunoprecipitation; UF: unfused myoblasts and F-fused mytoubes; TF: transcription factor; PcG: Polycomb Group.

## Authors' contributions

MLA jointly conceived the studies, designed and performed the experiments and drafted the manuscript. CK and GC participated in the ChIP-on-chip analysis. CK performed the bioinformatics on the ChIP-on-chip data. ID advised on the design of the ChIP-on-chip experiments and gave constructive comments on the drafting of the manuscript. MA conceived studies and participated in manuscript drafting. All authors have read and approved the final manuscript.

## Supplementary Material

Additional file 1 **Figures S1–S4 and Table ST1**. the data provided show expression data for *DES*, extra ChIP-on-chip data and PCR primer optimisation. Figure S1 shows light microscope images of unfused myoblast and fused myotubes cultures of human primary skeletal muscle cells. Figure S2 shows expression data for *DES *in expressing and non-expressing cells. Figure S3 gives more detailed genome browser maps of ChIP-on-chip data. Figure S4 shows the dissociation curves and DNA titrations for primers used in the real-time PCR analysis across the *DES *LCR and *DES*. This assessed the specificity of the PCR primer sets for their target sequence and their amplification efficiency. Table ST1 shows all primers used for microarray, real-time PCR, methylation restriction and bisulphite sequencing analysis. Click here for file
