# Supplementary material for: DNA methylation-histone modification relationships across the desmin locus in human primary cells
Source: BMC Mol Biol. 2009 May 27;10:51. doi: 10.1186/1471-2199-10-51 (PMC2695444; doi:10.1186/1471-2199-10-51)
Supplement: Additional file 1 — Figures S1–S4 and Table ST1. the data provided show expression data for DES, extra ChIP-on-chip data and PCR primer optimisation. Figure S1 shows light microscope images of unfused myoblast and fused myotubes cultures of human primary skeletal muscle cells. Figure S2 shows expression data for DES in expressing and non-expressing cells. Figure S3 gives more detailed genome browser maps of ChIP-on-chip data. Figure S4 shows the dissociation curves and DNA titrations for primers used in the real-time PCR analysis across the DES LCR and DES. This assessed the specificity of the PCR primer sets for their target sequence and their amplification efficiency. Table ST1 shows all primers used for microarray, real-time PCR, methylation restriction and bisulphite sequencing analysis. [file 1471-2199-10-51-S1.pdf]

S1

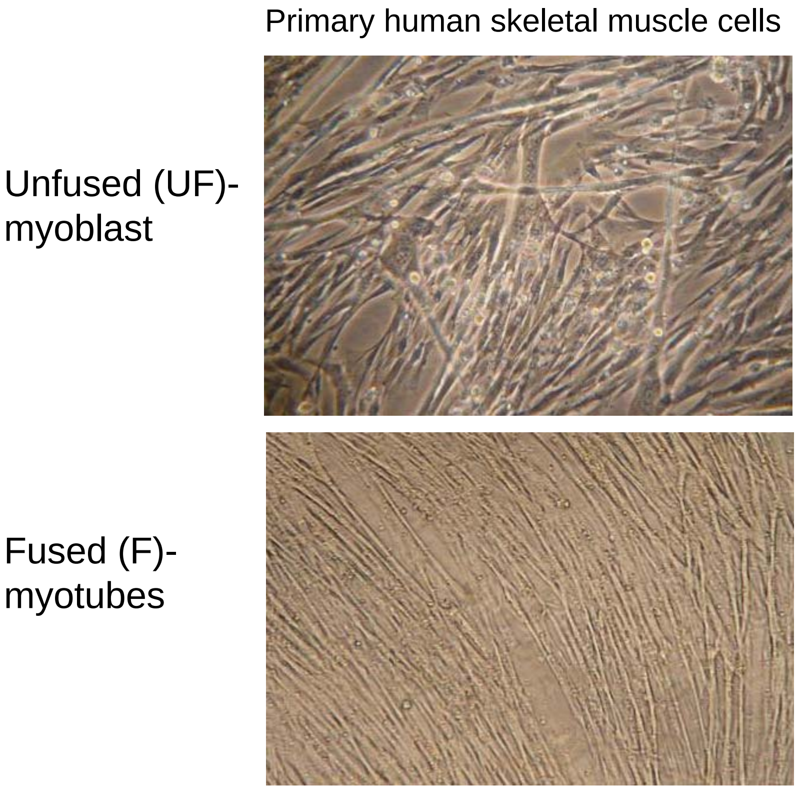

<u>Figure S1: Light microscope images of human primary unfused myoblasts and fused myotubes</u>. Mono-nucleated undifferentiated myoblasts (upper panel) can form multinucleated myotubes (lower panel) when grown to confluency and transferred into non-mitotic medium by reducing the fetal calf serum (FCS) concentration.

S2

A

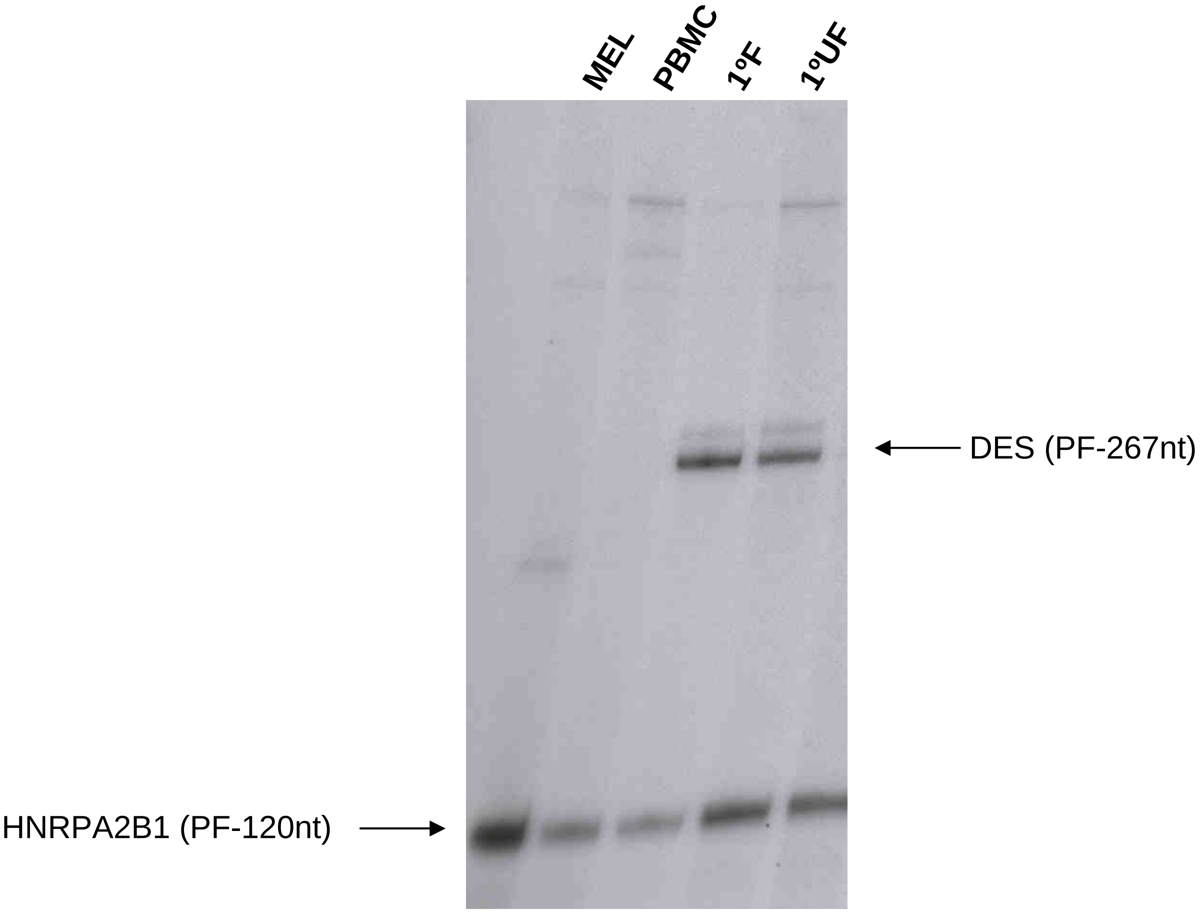

B

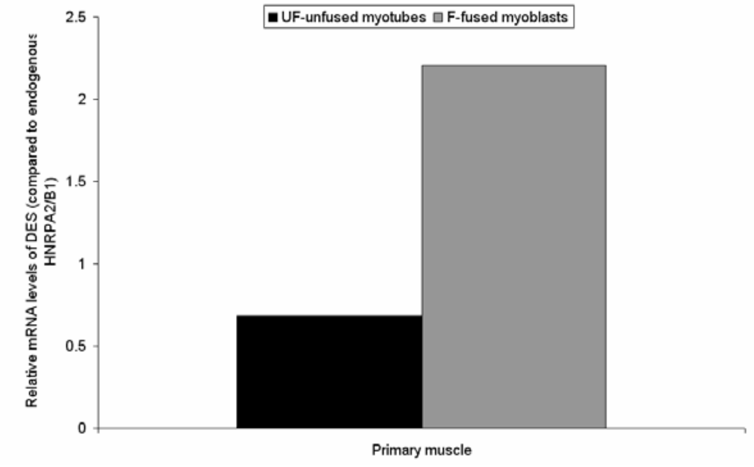

Figure S2: S1-Nuclease Protection Assay for *DES* expression in primary skeletal myoblast cell cultures. **(A)** Autoradiogram of S1-nuclease protection assay products after resolution on a denaturing polyacrylamide gel. Reactions were conducted on total RNA from murine erythroleukaemia (MEL) cells, human peripheral blood mononuclear cells (PBMCs), human primary adult skeletal myoblast (1°UF) and fused myotube (1°F) cultures. The end-labeled DNA probes gave S1-nuclease protected fragments (PF) of 267 nucleotides (nt) for DES mRNA and 120nt for HNRPA2B1. **(B)** *DES* expression was quantified from the phosphorimager scanned gel image using the ImageQuant © software (Version 5.1, GE Healthcare) and expressed as a ratio to HNRPA2B1 mRNA levels.

S3

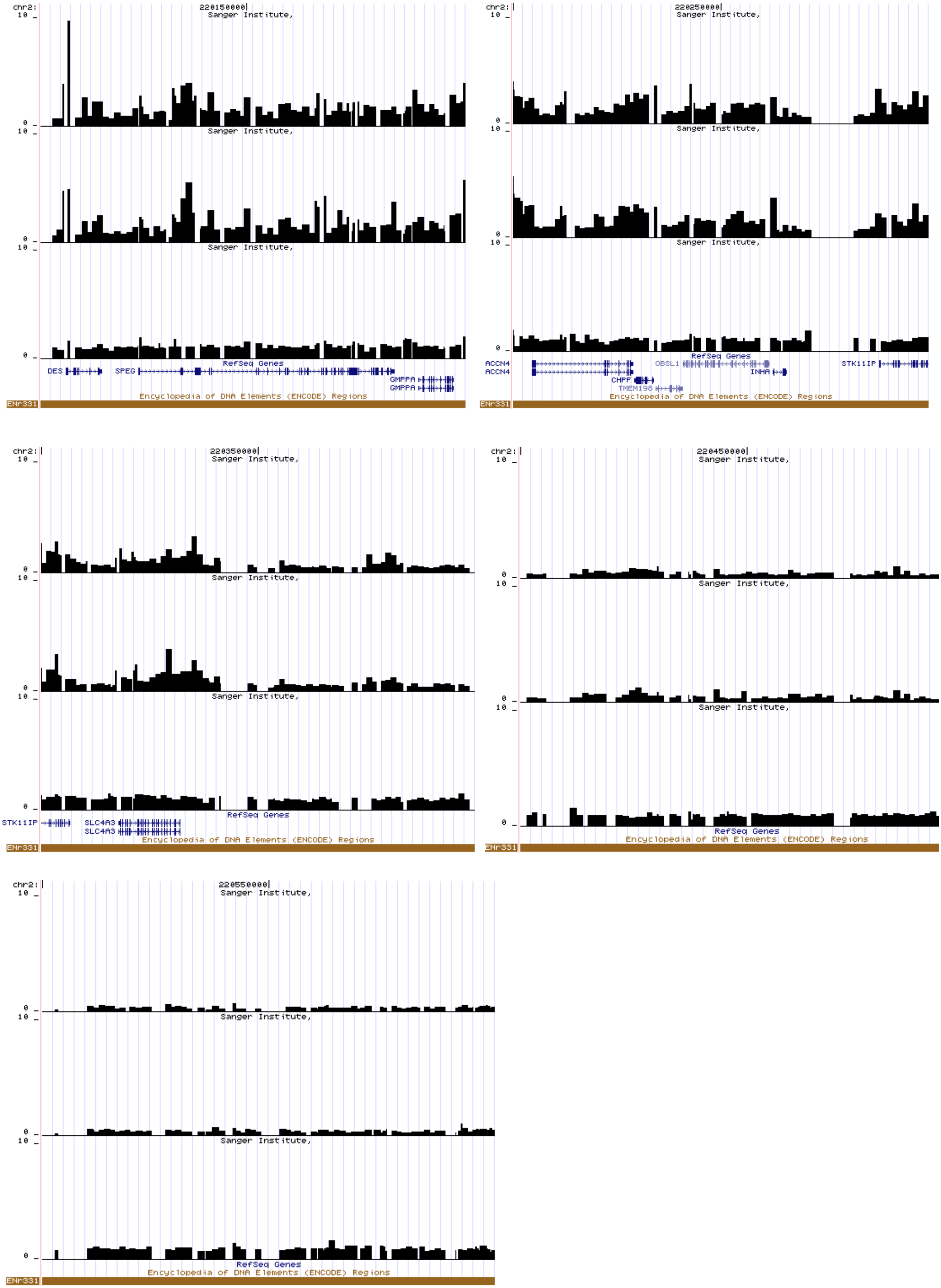
chr2:
220150000
Sanger Institute,
10
0
RefSeq Genes
DES
SPEG
GMPPA
GMPPA
Encyclopedia of DNA Elements (ENCODE) Regions
ENr331
220250000
ACCN4
ACCN4
OBSL1
INHA
CHPF
TMEM198
STK11IP
220350000
STK11IP
SLC4A3
SLC4A3
220450000
220550000

S3'

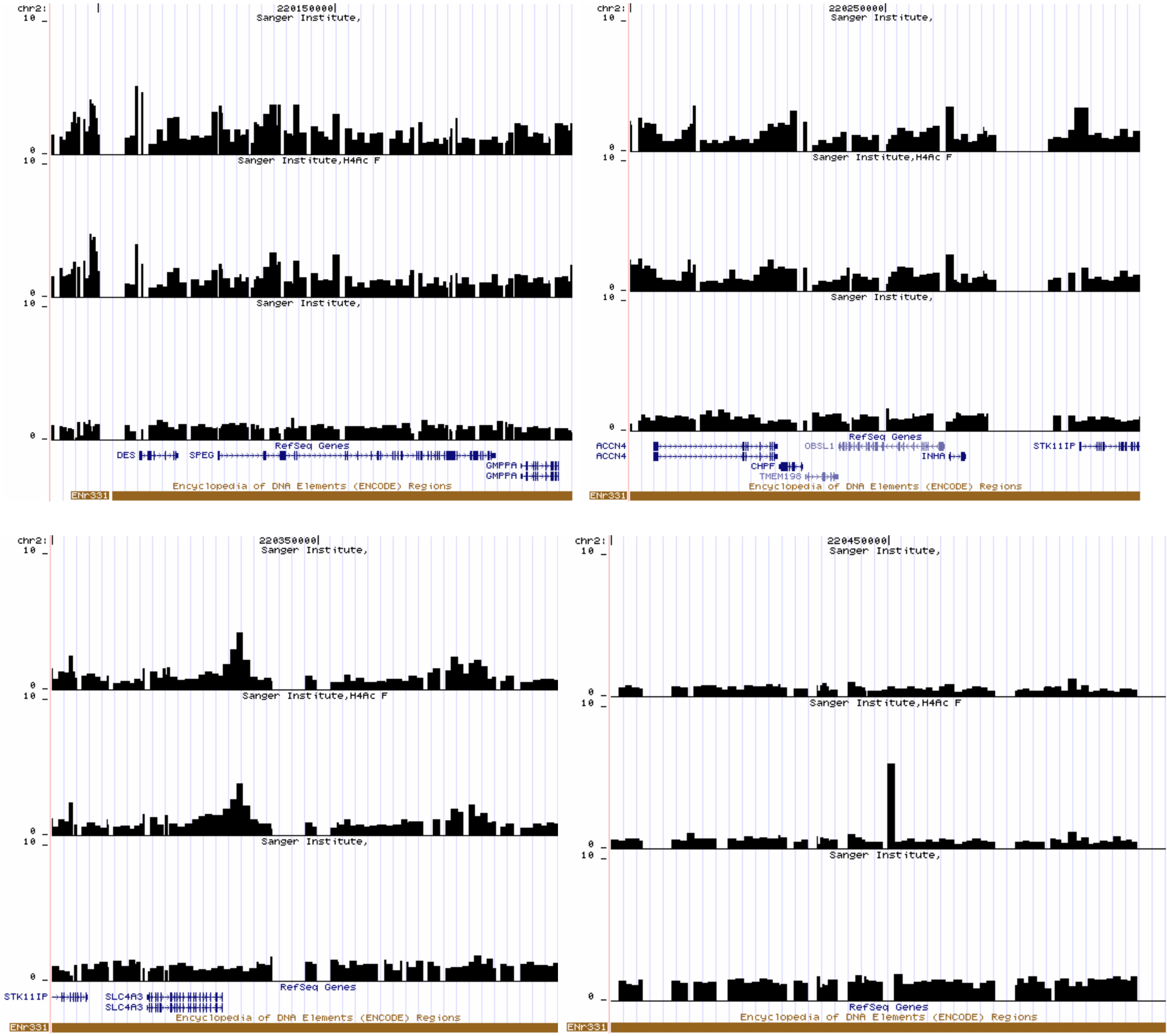

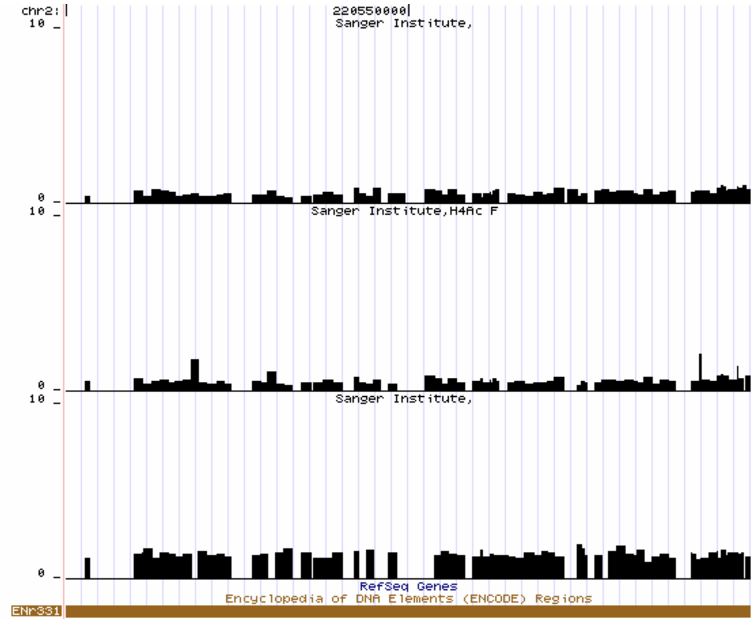

Figure S3: Microarray data for histone H3 and H4 acetylation across 500kb of human chromosome 2q35. A series of higher magnification maps illustrating the data from Figure 1 within the main manuscript.

S4

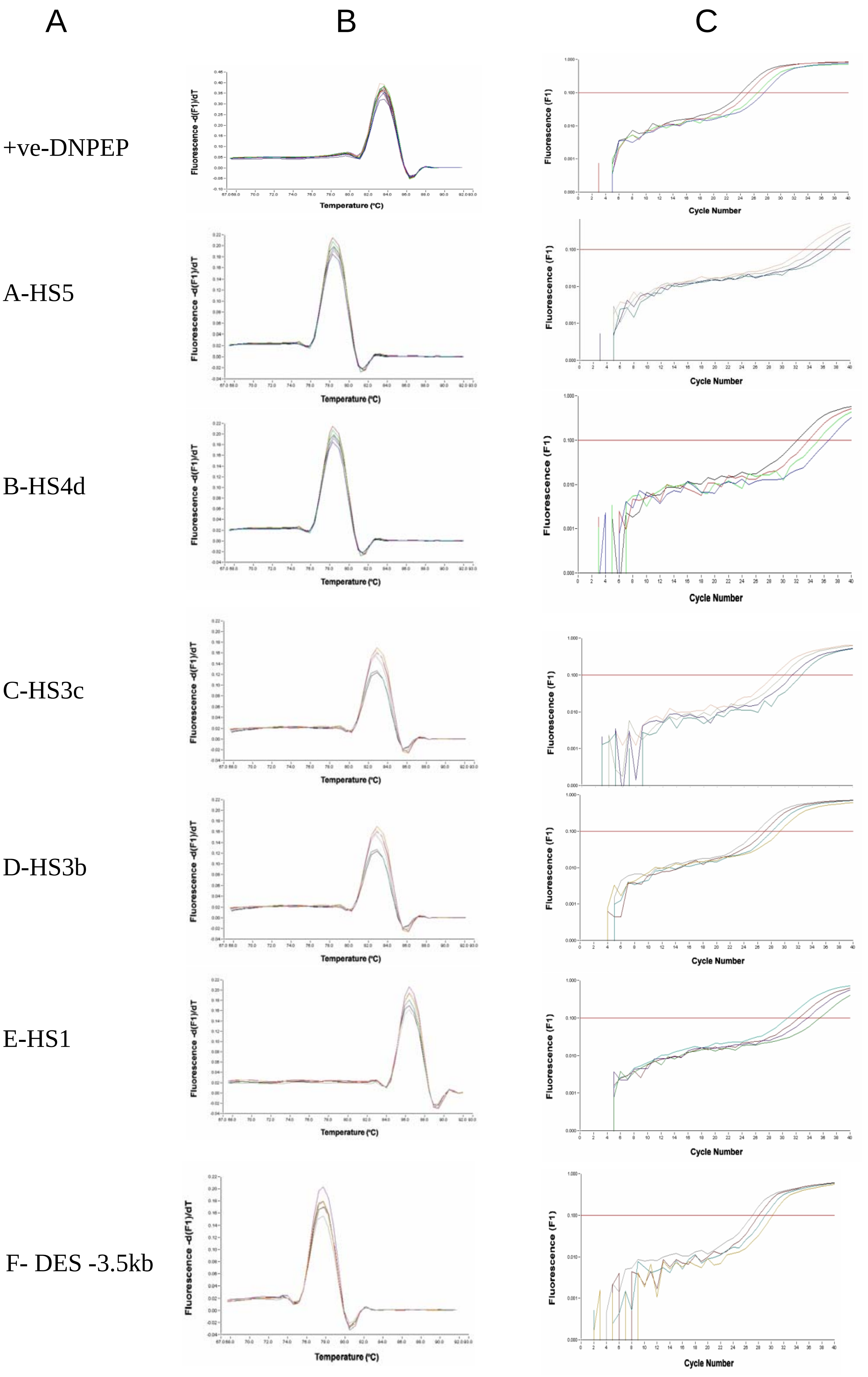
A
B
C
+ve-DNPEP
A-HS5
B-HS4d
C-HS3c
D-HS3b
E-HS1
F- DES -3.5kb
Fluorescence -d(F1)/dT
Temperature (°C)
Fluorescence (F1)
Cycle Number

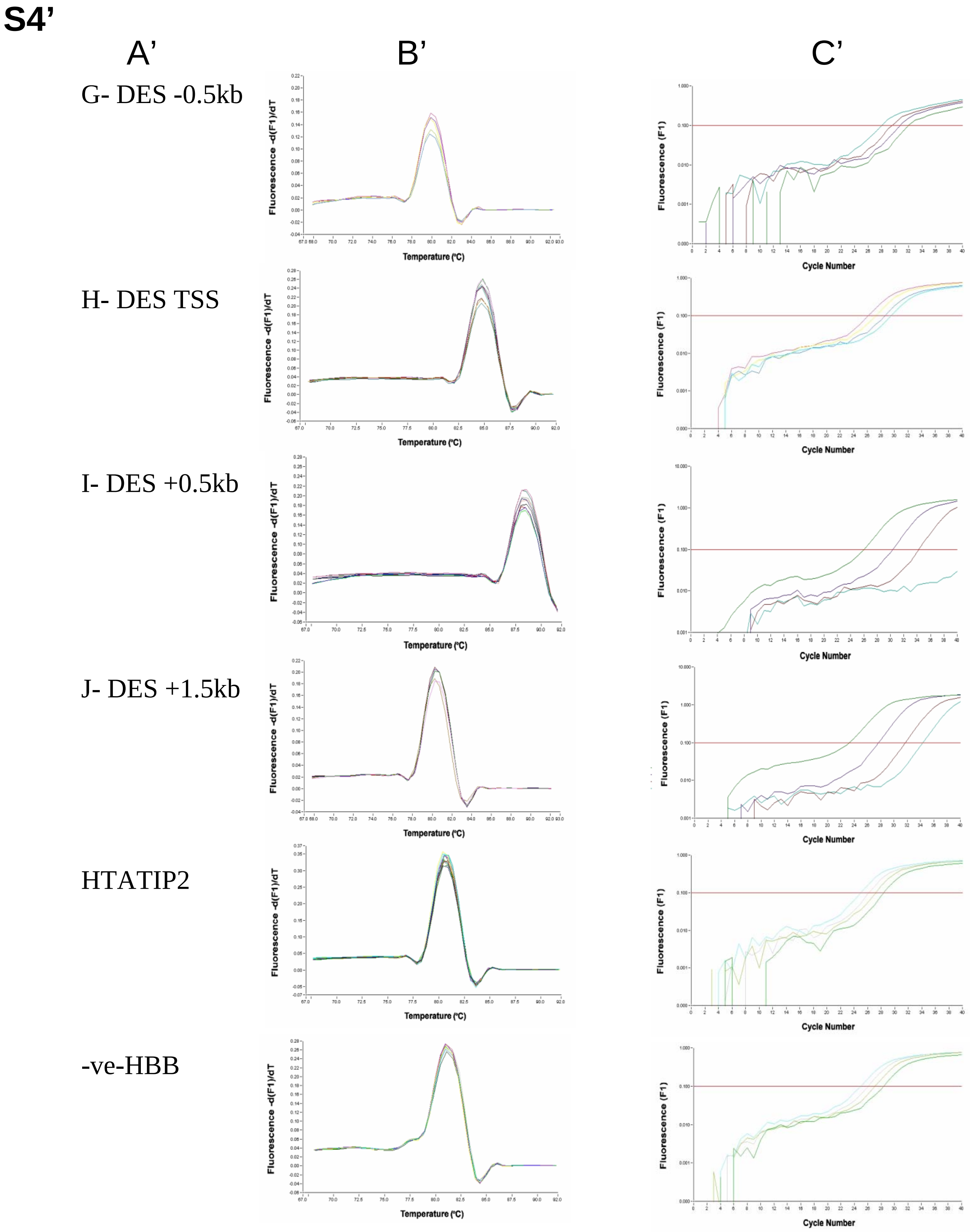

Figure S4: Melting curves and DNA titrations for primers used in the real-time PCR analysis across the *DES* LCR and *DES*. To assess the specificity of the PCR primer sets for their target sequence a melting curve programme was run and DNA titrations with input chromatin were performed on each primer set to evaluate their amplification efficiency. **(A and A')** Names of amplicons across the *DES* LCR and *DES* including the positive controls *DNPEP* and *HTATIP2* and the negative control *HBB*. Positions of amplicons given relative to the *DES* TSS are indicated for the primers which amplify *DES*. **(B and B')** The melting curves for all the amplicons. A single peak indicates PCR primers that are specific for their target sequence. **(C and C')** DNA titrations on input micrococcal nuclease (MNase) digested DNA were performed in order to examine their amplification efficiency so that primer sets could be compared against each other. All primer sets amplified the varying amounts of DNA with similar efficiency.

**Primers for tiling array**	
MLA1	5’-tgaccatgacagaactagacaggccccg-3’<br>3’- aaacagggagggtcgtgtcc-5’
MLA2	5’-tgaccatgacacgaccctccctgtttcc-3’<br>3’-gtgagcgttcgggattgatt-5’
stSG1510983	5’-accatggtctaggagtagaggacccagc-3’<br>3’-accatgacttagcagccaca-5’
stSG1510984	5’-tgaccatgagtgagccagagatggcatt-3’<br>3’-cccacccatagtcaggctta-5’
stSG1510985	5’-tgaccatgtaagcctgactatgggtggg-3’<br>3’-gtgtgcacctattgcttatgg-5’
stSG1510986	5’-tgaccatgaggtgcacacaaagctgaaa-3’<br>3’-cctccacagggcatttctta-5’
stSG1510987	5’-tgaccatgggtaagaaatgccctgtgga-3’<br>3’-gctctggctctgcaagactc-5’
stSG1510988	5’-tgaccatggagtcttgcagagccagagc-3’<br>3’-taggctccaacttctcccaa-5’
stSG1510989	5’-tgaccatgttgggagaagttggagccta-3’<br>3’-gcatcaggagtgtcccagat-5’
stSG1510990	5’-tgaccatgatctgggacactcctgatgc-3’<br>3’-ttctgctctgctcctcttcc-5’
stSG1510991	5’-tgaccatgtccatgcctagaccgtttct-3’<br>3’-ttatagcccagcccatcatc-5’
stSG1510992	5’- accatgtcagatgcttctcaaattcctc-3’<br>3’-aggctggcaatgacctcac-5’
stSG1510993	5’-tgaccatgggaggaagaggaggatgagc-3’<br>3’-gctgtgtgacattggactgg-5’
stSG1510994	5’- accatggggtcagagtagggaggtatgt-3’<br>3’-gcattaagaaaggtcccagg-5’
stSG1510995	5’-tgaccatgtcccaaagtgctgggattac-3’<br>3’-ggctaatctcacctcctccc-5’
stSG1510996	5’-tgaccatggagtccacaaagccctttca-3’<br>3’-tttggcttaagacattcggg-5’
stSG1510997	5’-tgaccatggaagatatggcccgaatgtc-3’<br>3’-cctgttgtgatcccagtgtg-5’
stSG1510998	5’-tgaccatgcacactgggatcacaacagg-3’<br>3’-tggctagtatgggcggatag-5’
stSG1510999	5’-tgtgaccatgcaaatgccctatgctggc-3’<br>3’-ccagcttctctcctttccct-5’
**Primers for high resolution nChIP**	
DNPEP	5’-gccaataaaaagctcgcccct-3’<br>3’-cccatggacattagccttattgct-5’
A (HS5)	5’-caatggtatctcatctagcaggattt-3’<br>3’-agtggttgtcagcacaggcaga-5’
B (HS4d)	5’-agccacacagagaagctgtaaa-3’

	3’-acagccaccaagagttgttca-5’
C (HS3c)	5’-tctccagggaagggccattt-3’<br>3’-cttcttggacacctctcccaa-5’
D (HS3b)	5’-tcccagcctgagtggcagca-3’<br>3’-gccaccctggataccagaata-5’
E (HS1)	5’-gtggattgctggctgagcctg-3’<br>3’-ggaatgtccagaggcggctgtt-5’
F (DES -3.5kb)	5’-cttaatctggaaatatttcaaacggaga-3’<br>3’-gtgtatgtgggatggtgcctt-5’
G (DES -0.5kb)	5’-ttaggaacaaggtggtttaggg-3’<br>3’-aggcttccctttagtttgag-5’
H (DES TSS)	5’-cgagctgctggacttctcac-3’<br>3’-cgaagcggtcattgagctcc-5’
I (DES +0.5kb)	5’-agggcccggcaccccaga-3’<br>3’-tttctccacatggggcaggaga-5’
J (DES +1.5kb)	5’-acgtggatgcagctactctag-3’<br>3’-ctcttcatgcactttcttaagg-5’
HTATIP2	5’-aaagtcacgctcattggccg 3’<br>3’- tcctgggggtccatttgagt-5’
-ve (HBB)	5'-cagtgcaggctgcctatcag-3'<br>5'-cccccagtttagtagttgga-3'
**Primers for HpaII methylation analysis**	
HS1<br>HS1	5’-ttcagccatgtgctcccaat-3’<br>3’-ggagctggagtgactgtaaaag-5’
DES	
DES+0.5<br>DES+0.5	5’-tggcaggacagcgggatctt-3’<br>3’-cacacaggtggaggacccttt-5’
DES+1.5<br>DES+1.5	5’-aggctgcaggaggagatt-3’<br>3’-cagcacatccaagctgggatt-5’
Quality control<br>chr2:219,973,389-219,974,059	5’- ctttctggggcatgaagacat-3’<br>3’- ttctctacttggcatccacga-5’
**Primers for amplification of bisulphite treated DNA**	
I (HS5)<br><br>I nested	5’-attagaatgtgagttttaggaggata-3’<br>3’- ccaacataaaaaaacctcatctctacta-5’<br>5’-aatgtatgtttggtgtatttgttgtgg-3’<br>3’- aaacacctataatcccaactactcaaa-5’
II (HS4)<br><br>II nested	5’- tagtgtggtttaggaggaggaagt-3’<br>3’- ttataaatcatcttccaatttcaaaatata-5’<br>5’ agtttggatttgatgtttattttt-3’<br>3’- cctaaataacctaaataactcacaa-5’
III (HS3b&c)<br><br>III nested	5’- ggtagtttgaggtaggagaattgttt-3’<br>3’- aaaacaaatactccctctaccctct-5’<br>5’- tgagggtgttttaggtatag-3’<br>3’- caaccaccctaaataccaaaa-5’

V (Intergenic region)	5’- aaataaatagaaaatagtaagtgtt-3’
	3’- acattcacattattatacaactat-5’
V nested	5’- tgyggaaaatagtatggtagtttt-3’
	3’- actccatatatattaaaaataaccct-5’
VI (5’DES)	5’- gttgttgttagggagatggttgggtt-3’
	3’- actatttatatccctcctaacatcaa-5’
VI nested	5’- ctcccctcccctaacaactacaaaatc-3’
	3’- agggggtaggagttatataaagttt-5’
VII (DES)	5’- ttaggtggaggtgtttattaattag-3’
	3’- aacaaaacaaaaccaaaaaccatac-5’
VII nested	5’- ggtagggtataggaggttaggtttg-3’
	3’- aataacccaatctctacccaaaaac-5’
